# Identification of a novel specific small-molecule melanocortin-2-receptor antagonist

**DOI:** 10.1530/EC-22-0338

**Published:** 2022-10-06

**Authors:** Rachel Forfar, Mashal Hussain, Puneet Khurana, Jennifer Cook, Steve Lewis, Dillon Popat, David Jackson, Ed McIver, Jeff Jerman, Debra Taylor, Adrian JL Clark, Li F Chan

**Affiliations:** 1 Centre for Therapeutics Discovery, LifeArc, Accelerator Building, Open Innovation Campus, Stevenage, UK; 2 Centre for Endocrinology, William Harvey Research Institute, Queen Mary University of London, London, UK

**Keywords:** MRAP, MC2R, adrenal, GPCR antagonism, drug discovery

## Abstract

The overproduction of adrenocorticotropic hormone (ACTH), in conditions such as Cushing’s disease and congenital adrenal hyperplasia (CAH), leads to significant morbidity. Current treatment with glucocorticoids does not adequately suppress plasma ACTH, resulting in excess adrenal androgen production. At present, there is no effective medical treatment in clinical use that would directly block the action of ACTH. Such a therapy would be of great clinical value. ACTH acts via a highly selective receptor, the melanocortin-2 receptor (MC2R) associated with its accessory protein MRAP. ACTH is the only known naturally occurring agonist for this receptor. This lack of redundancy and the high degree of ligand specificity suggest that antagonism of this receptor could provide a useful therapeutic strategy in the treatment of conditions of ACTH excess. To this end, we screened an extensive library of low-molecular-weight drug-like compounds for MC2R antagonist activity using a high-throughput homogeneous time-resolved fluorescence cAMP assay in Chinese hamster ovary cells stably co-expressing human MC2R and MRAP. Hits that demonstrated MC2R antagonist properties were counter-screened against the β2 adrenergic receptor and dose–response analysis undertaken. This led to the identification of a highly specific MC2R antagonist capable of antagonising ACTH-induced progesterone release in murine Y-1 adrenal cells and having selectivity for MC2R amongst the human melanocortin receptors. This work provides a foundation for the clinical investigation of small-molecule ACTH antagonists as therapeutic agents and proof of concept for the screening and discovery of such compounds.

## Introduction

The melanocortins, comprising the α-, β-, and γ-melanocyte-stimulating hormones (MSHs) and adrenocorticotropic hormone (ACTH), are a family of peptide hormones cleaved from the prohormone pro-opiomelanocortin that have a diverse range of physiological functions. As part of the hypothalamic–pituitary–adrenal axis, ACTH is secreted by pituitary corticotrophs into the systemic circulation under the control of hypothalamic corticotropin-releasing hormone (CRH) and arginine vasopressin. ACTH then acts on the adrenal glands to produce glucocorticoids and cortisol in humans. Cortisol negatively feeds back at the level of the pituitary gland and hypothalamus to inhibit further production and release ACTH.

The melanocortins act through five known melanocortin receptors (MC1R–MC5R) of the class A seven transmembrane G-protein-coupled receptor family (GPCR) which, when activated, stimulate the production of intracellular cAMP (1). ACTH is the only endogenous agonist for MC2R, also known as the ACTH receptor. MC2R is the smallest human GPCR and, although it shares nearly 50% homology with other MCR subtypes, MC2R is unique among MCRs due to its absolute selectivity for ACTH. In contrast, MC1R, MC3R, MC4R, and MC5R can be activated by α-, β-, and γ-MSH and ACTH. The lack of redundancy and high degree of ligand specificity, restricted tissue distribution pattern (primarily expression within the zona fasciculata of the adrenal cortex), and the fact that the MC2R is wholly responsible for the production of glucocorticoids suggest that MC2R may represent a good target for pharmacological intervention. This molecular and physiological specificity is highlighted by the condition familial glucocorticoid deficiency type 1 (FGD1; OMIM ♯202200) caused by loss of function mutations in MC2R that lead to isolated cortisol deficiency (2).

Technically, the ability to screen for MC2R modulators was hampered by the lack of MC2R trafficking and signalling in heterologous cell lines of non-adrenal origin (3). This was only made possible through the discovery of the melanocortin-2-receptor accessory protein (MRAP), which is essential for MC2R trafficking and binding to ACTH (4). MC2R is unusual in that it is absolutely dependent on MRAP for cell surface expression and function. MRAP expression is also highly restricted to the adrenal cortex, and loss of function mutation in MRAP leads to familial glucocorticoid deficiency type 2 (FGD2; OMIM #607398) (4). As a result, the MC2R/MRAP complex provides an ideal target for a selective antagonist as its biological effect should be highly specific.

Elucidating the molecular basis of MCR ligand binding and signalling is therefore a prerequisite to the development of selective MC2R agonists and antagonists for the treatment of adrenal disorders. Investigation of the melanocortin peptides shows the conservation of the core tetrapeptide sequence, His-Phe-Arg-Trp (5, 6). This sequence is crucial for MCR binding and second messenger activation. This core sequence has been utilised for the design of α-MSH peptide analogues, resulting in the development of potent and highly selective peptide agonists and antagonists for each of the melanocortin receptor subtypes. A further sequence Lys-Lys-Arg-Arg at positions 15, 16, 17, and 18 (the address sequence) within ACTH is essential for binding and second messenger activation of the MC2R–MRAP complex by ACTH (6). None of the other melanocortin peptides, α-MSH, β-MSH, and γ-MSH, possesses the address sequence.

With the discovery of MRAP, much progress has now been achieved with recombinant cell lines that fully reproduce dose–response curves of ACTH-induced cAMP production (4, 7). This allowed the identification of MC2R peptide antagonists (8, 9, 10). Specific advantages of a small molecule over a peptide antagonist include improved bioavailability and the option of oral administration. Metformin at a concentration of 10 mM has been recently shown to inhibit the activation of an MC2–MRAP complex by ACTH, although the biological relevance of this at these high concentrations is unclear (11).

The therapeutic potential for pharmacological blockade of the MC2–MRAP complex has been reviewed recently and is likely to be of value in managing conditions of ACTH excess (6). Conditions include Cushing’s disease where pituitary adenoma removal is unsuccessful, ectopic ACTH syndrome from tumours such as small cell lung cancers, and congenital adrenal hyperplasia (CAH) where ACTH drives excess androgen production (6, 12, 13). Therapies that aim to suppress ACTH and its actions are being pursued, including monoclonal antibodies against ACTH (14) and CRH receptor 1 antagonists with the latter in clinical trials for the treatment of ACTH-driven androgen excess in CAH (15). While a number of chemical inhibitors of cortisol synthesis are used as an adjunct to surgery to control cortisol levels, at present there is no effective medical treatment that directly blocks the action of ACTH at the MC2R–MRAP complex. In the present study, a compound library high-throughput screen was performed to identify MC2R antagonists. Candidate antagonist pharmacology was profiled against human and murine MC2R, and selectivity was confirmed with respect to other human MCRs. We show that a non-peptide small molecule, MCR2-4788, is a novel, selective MC2R/MRAP antagonist.

## Materials and methods

### Materials

Isoproterenol, α-MSH, BSA, and 3-isobutyl-1-methylxanthine (IBMX) were purchased from Sigma-Aldrich. ACTH 1-24 was purchased from Cambridge Research Biochemicals (Cleveland, UK). Homogeneous time-resolved fluorescence (HTRF) cAMP dynamic 2 assay kits were from Cisbio (Codolet, France). Progesterone ELISA kit was purchased from Cayman Chemical. GeneBLAzer® MC2R-CRE-*bla* CHO-K1 (containing the human MC2R (accession number NM_000529.1) and the MRAP (accession number NM_178817.4)) and GeneBLAzer® MC3R CRE-*bla* CHO-K1 cells were purchased from Invitrogen. CHO-K1, HEK293, and Y-1 cells were purchased from ATCC (LGC Standards, Teddington, UK). DMEM, dialysed fetal bovine serum (FBS), non-essential amino acids (NEAA), HEPES, blasticidin, zeocin, hygromycin, and Lipofectamine 2000 transfection reagent were all purchased from Invitrogen.

The eight chemical libraries used in our screening were as follows: LOPAC1280 (Sigma-Aldrich), HitFinder (Maybridge, Cornwall, UK), bioactive compounds (InterBioScreen, Moscow, Russia), BBP-2080 library (BioBiopha, Kunming, China), MEGxm and MEGxp (Analy-Ticon Discovery, Potsdam, Germany), Prestwick chemical library (Prestwick, Illkirch, France), Tocriscreen Total (Tocris, Bristol, UK), and GPCR-targeted library (ChemDiv, San Diego, CA, USA).

### Cell line generation and culture

CHO cells stably expressing the MC2R and MRAP were grown in a monolayer in DMEM supplemented with 10% dialysed FBS, 0.1 mM NEAA, 25 mM HEPES, 5 μg/mL blasticidin, 100 μg/mL zeocin, and 600 μg/mL hygromycin. The CHO-K1 cells were grown in DMEM with 10% FBS and transiently transfected (in-house) with 30 μg of β2-adrenergic receptors (ADRβ2) using Lipofectamine 2000 transfection reagent according to manufacturer’s guidelines. HEK293 cells were grown in a monolayer in DMEM supplemented with 10% FBS. Lipofectamine 2000 transfection reagent was used to transiently transfect HEK293 cells (in-house) with 6.6 μg pcDNA5/FRT-mMc2r and 13.3 μg pcDNA6-mMrap-HA. Y-1 cells were maintained in Ham’s F-12K, Kaighn’s modified medium supplemented with 2.5% FBS, and 15% horse serum. Lipofectamine 2000 transfection reagent was used to transiently transfect HEK293 cells (in-house) with 20 μg pSNAP-MC1 or pSNAP-MC5. HEK293 cells stably expressing SNAP-MC4 (created in-house; 20 μg of DNA was transfected and clonal cells selected with 800 μg/mL G418 in the medium) were grown under the same condition as native HEK293 cells, except for the supplementation of 200 vg/mL G418 in the medium. CHO cells stably expressing the MC3 receptor were grown in a monolayer in DMEM supplemented with 10% dialysed FBS, 0.1 mM NEAA, 25 mM HEPES, 5 μg/mL blasticidin, and 600 μg/mL hygromycin. All cell lines were grown at 37°C in the presence of 5% CO_2_ and 95% humidity. Cells were passaged prior to 90% confluency. Cells were expanded to be used as assay-ready aliquots and cryopreserved in FBS/10% DMSO at a density of 10–20 million cells per millilitre until the day of use. All cell lines were regularly checked for mycoplasma (Lonza) and were negative.

### HTRF cAMP accumulation assay

HTRF cAMP dynamic 2 assay kits, obtained from Cisbio, are based on a competitive immunoassay using cryptate-labelled anti-cAMP antibody and d2-labelled cAMP. Experiments were performed 48 h after transfection, or with stable cell lines expressing the relevant receptors, according to the manufacturer’s instruction. In brief, cells were harvested and resuspended to a density of 1.5 × 10^6^ cells/mL in assay buffer (calcium and Mmgnesium-free PBS), 5 mM HEPES, 0.1% BSA, 0.5 mM IBMX). The cell suspension (7500 cells in 5 μL) was dispensed into each well of a 384-well, white, low-volume plate (Greiner Bio-One Ltd, Stonehouse, UK). PBS (2.5 μL) containing chemical compound with indicated concentrations and the phosphodiesterase inhibitor IBMX with a final concentration of 200 mM were added, and the plate was incubated at room temperature for 30 min. Stimulation was applied by 2.5 μL of ligand or assay buffer (without IBMX) for 30 min at room temperature to facilitate cAMP accumulation. Lysis buffer (10 μL) containing d2-cAMP (a fluorescent-conjugated cAMP) and anti-cAMP antibody was then added. After 60-min incubation at room temperature in the dark, HTRF signal was detected with a PherastarPlus plate reader (BMG). Fluorescent emission at 620 and 665 nm was measured, using excitation of 337 nm.

### Progesterone release assay

Progesterone ELISA kit (Cayman Chemicals) is a competitive assay that uses an enzyme immunoassay (EIA) solid-support format to quantify the amount of progesterone in samples. The assay is based on the competition between progesterone and a progesterone acetylcholinesterase conjugate, or progesterone tracer, for a limited number of progesterone-specific rabbit antiserum-binding sties. The concentration of progesterone tracer is constant, while the concentration of progesterone in the samples varies; therefore, the amount of progesterone tracer that can bind to the rabbit antiserum is inversely proportional to the concentration of progesterone in the well.

Y-1 cells were harvested and resuspended to a density of 0.1 × 10^6^ cells/mL in the culture medium. The cell suspension (10,000 cells in 100 μL) was dispensed into each well of a 96-well black tissue culture-treated poly-d-lysine-coated plate (BD Biosciences, Erembodegem, Belgium). Cells were incubated overnight at 37^o^C to adhere to the plates. The following day the media was aspirated from the wells, and cells were stimulated for 2 h with a range of concentrations of ACTH made up in serum free media ± test compounds to determine antagonism, or forskolin as a positive control. Conditioned media was taken and manufacturer’s instructions were followed throughout to quantify the amount of progesterone released from Y-1 cells. Conditioned media was diluted 20-fold in serum-free media, and 50 μL/well were added to the progesterone EIA plate. Optimal read time for EIA colour development was 60 min.

### Statistics and data analysis

Assays were performed on 3 days, and each condition was tested in triplicate (*n*  = 3). s.d. between the replicates is represented by the error bars. The HTRF ratio (665 nm/620 nm) of raw florescence intensity values was calculated within each well and, where appropriate, expressed relative to high (100%: typically EC100 of stimulus or EC80 for antagonist assays) and low (0%; EC0) controls. Standard statistical analyses were conducted to calculate Z’ and variance around high and low controls, to support hit identification, activity cut-off calculation, and standard measures of HTS robustness (IDBS Abase, Surrey, UK).

## Results

### Screening of small-molecule candidate compounds

The library of ~200,000 non-peptide small molecules was screened using the HTRF^TM^ cAMP accumulation assay kit, CHO-MC2R-MRAP cells, and a 10 μM concentration of candidate compound (single-shot screening). Seven hundred and ninety-three candidate compounds were identified that inhibited ACTH-stimulated cAMP accumulation by at least 25% on a single-shot assay. These candidate compounds were subjected to a counter screen against another Gs-coupled receptor, the β2 adrenergic receptor (β2-AR) transiently expressed in CHO cells, in order to identify candidate compounds specific for MC2R. The β2-AR agonist isoproterenol was used for stimulation. Of the 793 candidate compounds, 208 were found to be capable of lowering the activity of MC2R by 50% or more whilst having little to no effect on β2-AR. These were further profiled.

### Hit confirmation

**Figure 1:**
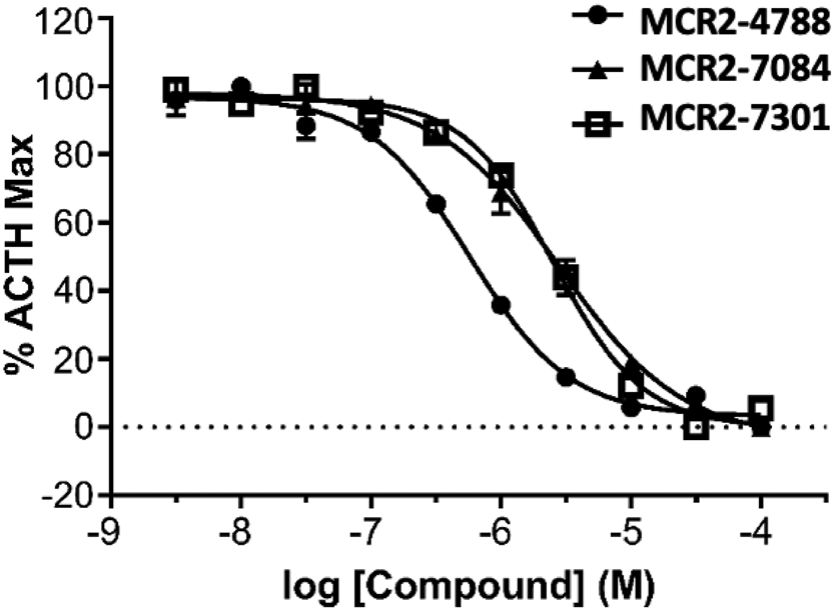
Hit compound confirmation and inhibition curves of compounds. Hit compounds from the high-throughput screen were confirmed if they were able to inhibit the MC2 receptor stimulated with an EC80 of ACTH in a concentration-dependent manner. Exemplar compounds shown are MCR2-7084, MCR2-7301, and MCR2-4788. Of note is MCR2-4788 which gave a pIC50 value of 6.2 and thus showed the greatest potential as a small-molecule antagonist of MC2R and MRAP.

Hits were reconfirmed in experiments using a single concentration of 10 μM. Subsequent *silico* triage, based on standard physico-chemical properties and drug-like filter criteria, reduced the hits to 59 compounds of interest. Further characterisation of these triaged hits was completed by compound concentration response curves (CRCs) showing inhibition of accumulation of cAMP produced by the MC2R/MRAP complex stimulated with ACTH at EC80. pIC50 values ranged from 6.2 to >4 (exemplar compounds shown in Fig. 1). MCR2-4788 gave a pIC50 value of 6.2 and therefore showed the greatest potential as a small-molecule antagonist of the MC2R/MRAP complex.

### Antagonist characterisation

**Figure 2:**
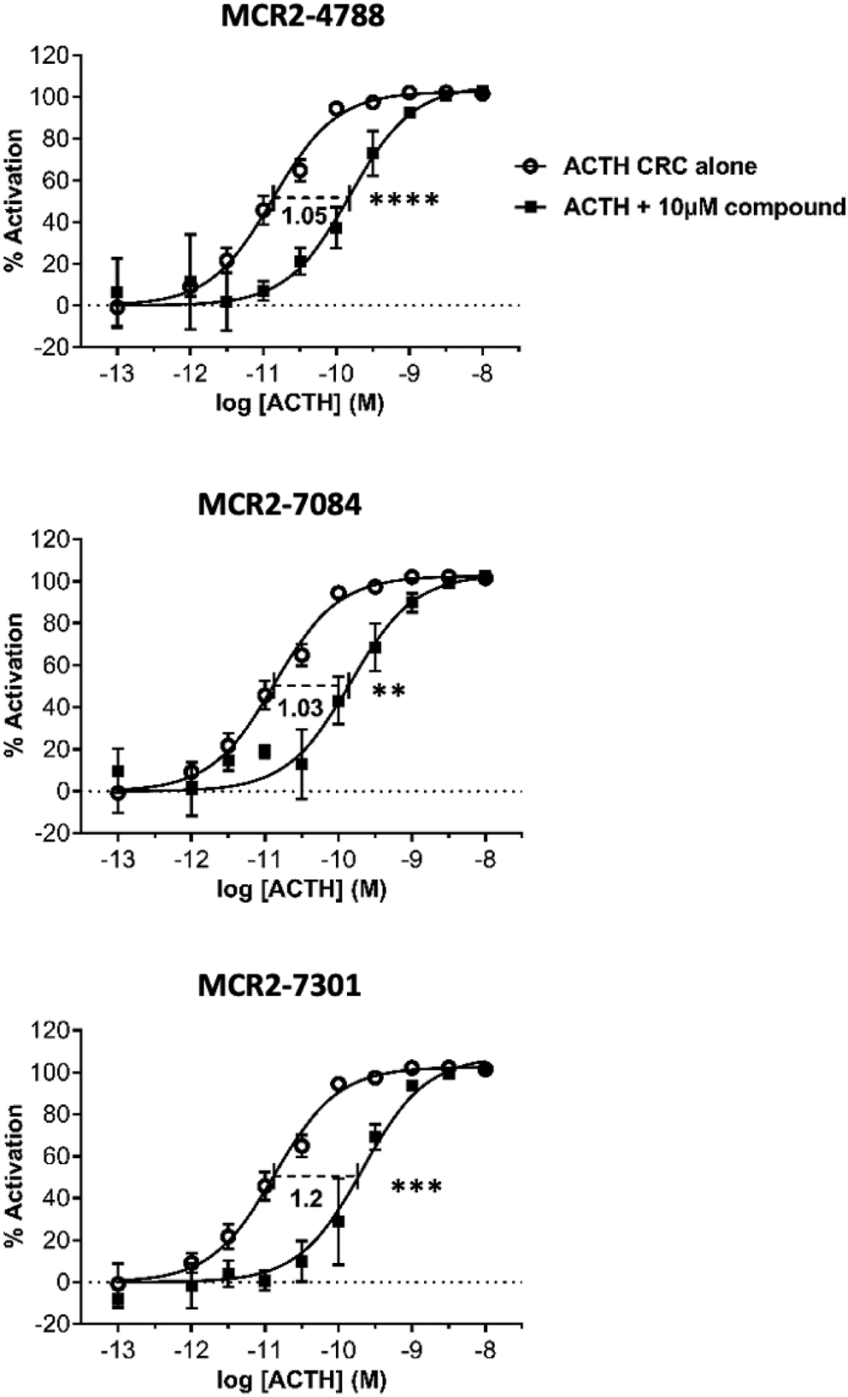
Antagonist characterisation. Distinct shifts were observed in the ACTH CRCs upon addition of the candidate compounds. Three compounds caused at least unit log shift in the values for half maximal effective concentration (EC50). The assay was performed on three separate days, and each condition was tested in triplicate (*n*  = 3). The nine replicates obtained confirmed reproducibility. s.d. between the replicates is represented by the error bars. Statistical significances between the LogEC_50_ levels are denoted graphically. **P < 0.01, ***P < 0.01 and ****P < 0.001.

**Figure 3:**
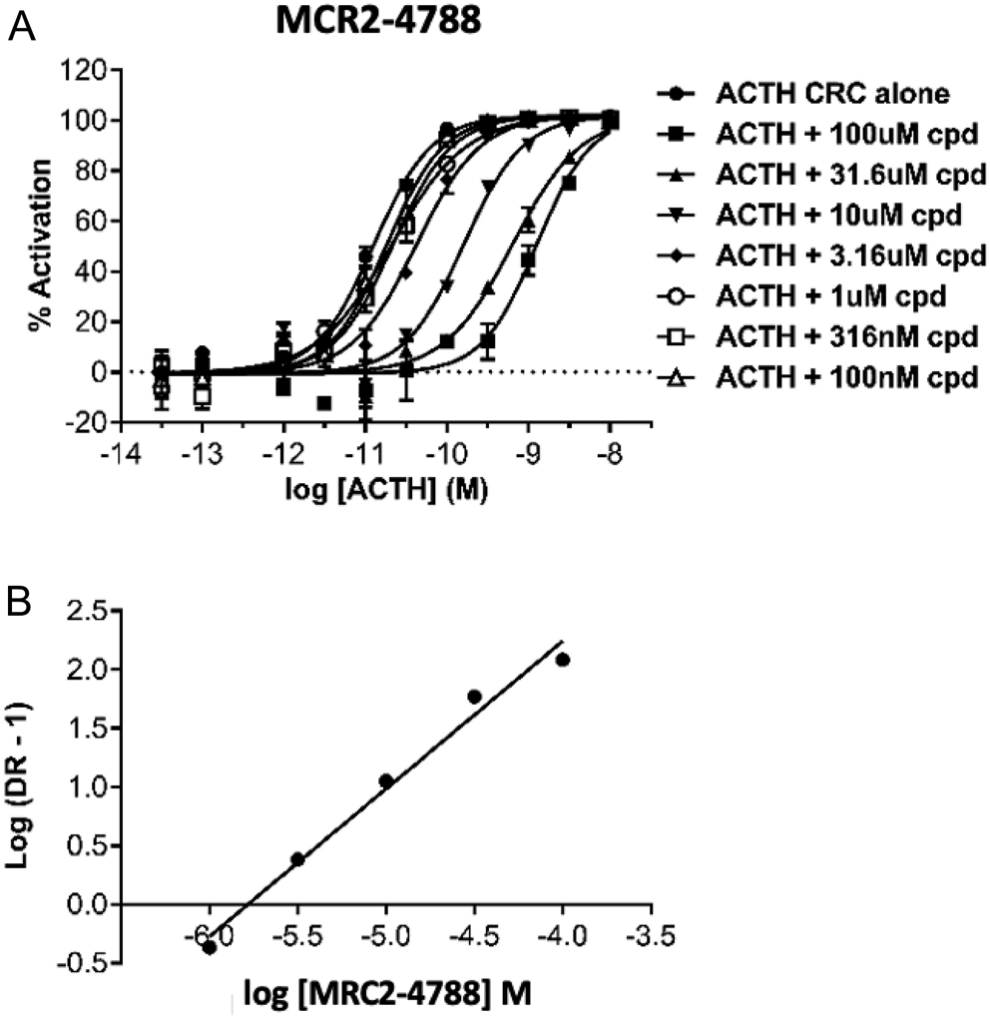
Antagonist characterisation. Functional activity of MCR2-4788 in cAMP accumulation assay. A, concentration-dependent stimulation of cAMP accumulation response in CHO-K1 cells expressing human MC2R and MRAP by ACTH in the presence of increasing concentrations of MCR2-4788. The data were derived from HTRF measurement and normalised to percentage of activity based on the effect of 100 nM ACTH. Each data point is mean ± s.d. (*n*  = 4). B, Schild plot of the data from A. Dose ratio is the ratio of the ACTH EC50 value in the presence of MCR2-4788 divided by the EC50 value in the absence of the antagonist. Calculated pA_2_ = 5.7.

Each candidate MC2R antagonist was further evaluated for its effect at 10 μM on the dose–response curve of ACTH in CHO-MC2R-MRAP cells using the HTRF cAMP assay. Three compounds consistently shifted the ACTH CRC by at least a log unit to the right (Fig. 2). A wider range of compound concentrations were tested (from 100 μM to 100 nM) to enable Schild regression analysis. All three antagonist candidates showed results consistent with a competitive nature of antagonism whereby the curves were consistently shifted to the right without reaching a maximal shift (indicative of allosteric modulation) or a drop in the efficacy (indicative of non-competitive antagonism), an example of which is presented for MCR2-4788 (Fig. 3). The calculated pA_2_ value for MCR2-4788 was 5.7, while the other two compounds tested MCR2-7084 and MCR2-7301 gave pA_2_ values of 5.8 and 5.9, respectively (see Table 1).

**Table 1:** pA2 values of tested hits Schild analysis of the three hit compounds revealed competitive mode of antagonism with pA2values shown.

Compound	pA_2_
MC2R-4788	5.7
MC2R-7084	5.8
MC2R-7301	5.9

### Antagonism of endogenous MC2R/MRAP in Y1-adrenocortical cells

**Figure 4:**
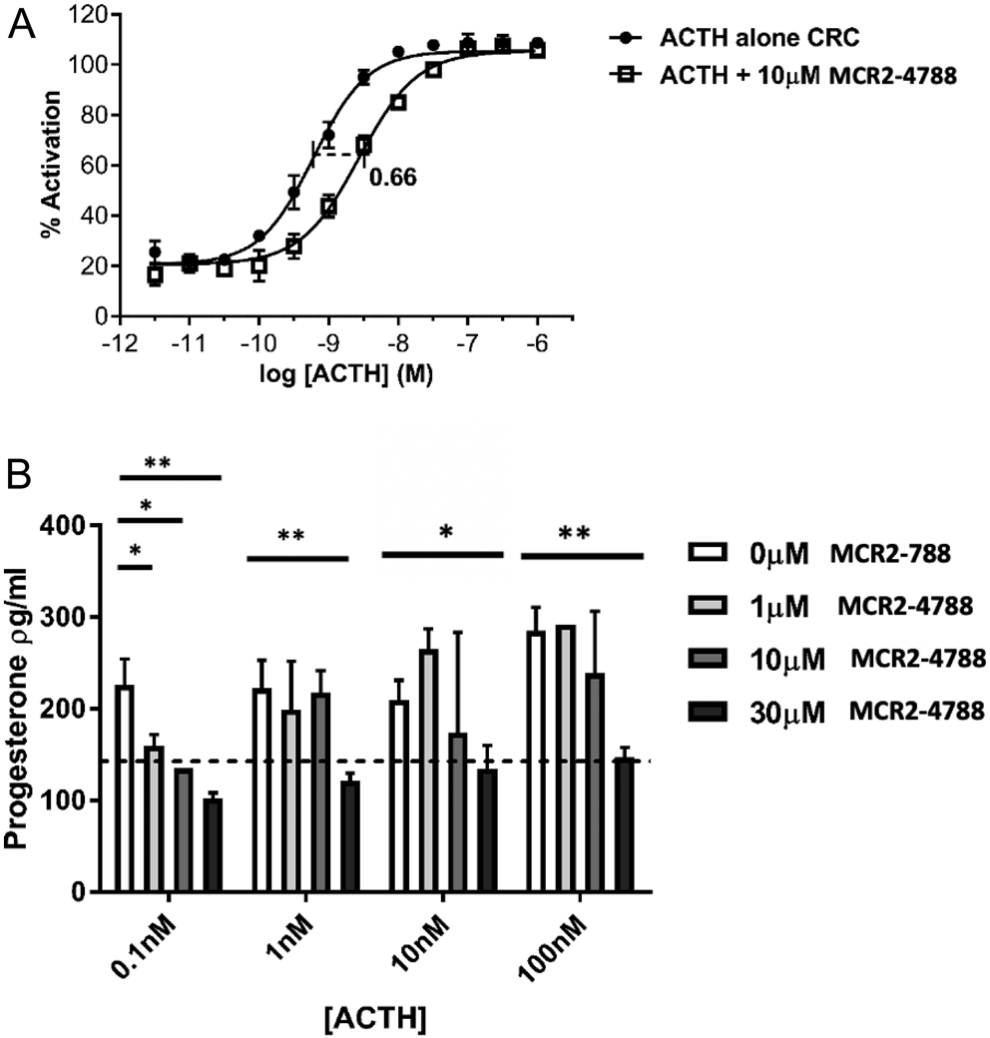
Antagonism of ACTH-induced cAMP generation and progesterone production in adrenal cells. (A) MCR2-4788 demonstrated antagonism of ACTH-induced cAMP accumulation in murine Y-1 adrenal cells, while MCR2-7301 and MCR2-7084 did not (negative data not shown). (B) MCR2-4788 inhibited ACTH-induced progesterone release in murine Y-1 adrenal cells. Progesterone release in Y-1 cells was induced by ACTH at 100 nM, 10 nM, 1 nM, and 100 pM concentrations, each in the presence of no candidate MCR2-4788, and 1 μM, 10 μM, and 30 μM of the MCR2-4788. The mean unstimulated baseline level of progesterone release is indicated by the dashed line (142.81 pg/mL +/–6.309). The assay was performed on 3 days, each condition being tested in triplicate (*n*  = 3). Error bars represent s.d. Statistical significance between each bar is shown graphically. *P < 0.05, **P < 0.01.

Initial profiling of the compounds was carried out using a commercially purchased recombinant cell line over-expressing human MC2R and MRAP. Ideally, effects should be observed in cell lines more representative of the adrenal cortex; therefore, the murine Y-1 cell line derived from an adrenocortical tumour, and with endogenous levels of MC2R and MRAP expression, was obtained. By HTRF cAMP assay, MCR2-4788 also shifted the ACTH CRC to the right by 0.66 of a log in Y-1 cells (ACTH pEC_50_ alone = 9.22; ACTH + 10 μM MCR2-4788 pEC_50_ = 8.56), demonstrating antagonism in a more physiologically relevant system (Fig. 4A), while the other two hit compounds, MCR2-7084 and MCR2-7301, demonstrated no rightward shift in this cell line (data not shown). The murine Y-1 cell line does not express 21-hydroxylase so is unable to produce corticosterone (16, 17). However, progesterone, an alternative marker of steroidogenesis that accumulates in response to ACTH stimulation, is an alternative and can be detected by ELISA. MCR2-4788 inhibited ACTH-induced progesterone release in this more physiologically relevant system (Fig. 4B). With a range of ACTH concentrations to stimulate progesterone release, there was antagonism seen with MCR2-4788 in a concentration-dependent manner. Progesterone release was significantly reduced at all doses of ACTH tested when a concentration of 30 μM MCR2-4788 was added. Statistically significant differences in progesterone release were also noted when Y-1 cells were stimulated at 100 pM concentration in the presence of 1 μM and 10 μM MCR2-4788. At 30 μM MCR2-4788, progesterone release was close to or at the baseline level of progesterone production, which is represented by the dashed line in Fig. 4B.

### Melanocortin receptor selectivity

**Figure 5:**
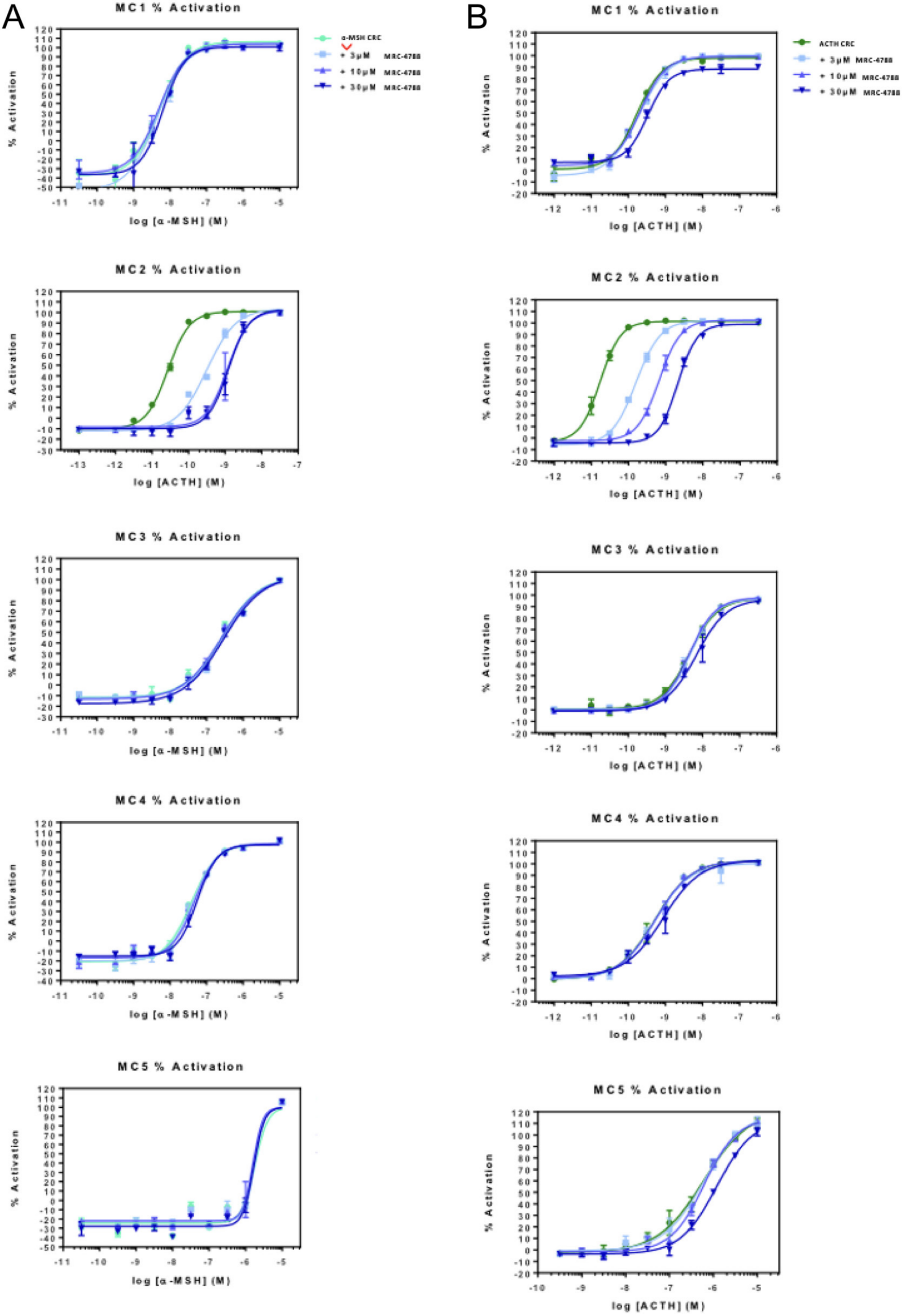
MCR selectivity against α-MSH and ACTH. (A) The α-MSH dose responses for cells expressing MC1R, MC3R, MC4R, and MC5R were not affected by MCR2-4788, whereas the ACTH dose responses for MC2R-expressing cells underwent a rightward shift. (B) Dose–response curves for ACTH at MC1R, MC2R, MC3R, MC4R, and MC5R, with ACTH alone and with the three concentrations of lead compound 4 indicated (*n*  = 3). MCR2-4788 did not antagonise MC1R, MC3R, MC4R or MC5R when stimulated with α-MSH or ACTH at the concentrations tested.

Now that a lead compound with functional antagonism effects on the MC2R had been identified, the compound was screened at the other melanocortin receptors in the family to assess how selective it was as an antagonist. First, α-MSH was used as the endogenous ligand to stimulate the closely related melanocortin receptor family members (Fig. 5A), while ACTH was still used for the cells expressing the MC2R since this is the only ligand able to stimulate this receptor. In the presence of MCR2-4788 at concentrations 3 μM, 10 μM, and 30 μM, there was only parallel rightward shifts of the ACTH curve seen, and no shifts were observed with α-MSH at any of the other MCR subtypes (Fig. 5A). Further studies are needed to investigate any potential antagonism at these other MCR family members, and the strong selectivity of MCR2-4788 as an antagonist at the MC2R was confirmed when using ACTH as the stimulating ligand (Fig. 5B).

## Discussion

In this study, we show the identification of MCR2-4788 from HTS and demonstrate that it is a novel non-peptide small-molecule antagonist at the MC2R. This small-molecule antagonist shows specificity against the β2-AR and selectivity over the other melanocortin receptor subtypes. Additionally, this molecule exhibits functional antagonism in the Y-1 adrenocortical cell line, both with cAMP response and the more physiologically relevant progesterone release response. Since the aim of the project was to find MC2R–MRAP complex antagonists suitable for clinical applications, we demonstrate the sequence of assays we undertook designed to rapidly identify potential antagonists and to reveal characteristics of their mode of antagonism.

Since the identification of MRAP as the critical missing MC2R accessory factor for the functional expression of the receptor in adrenal cells, this has enabled the large-scale drug screening, leading to the identification of a number of peptide MC2R antagonists (8, 9, 10). Furthermore, there is growing awareness of the unmet need in some conditions of ACTH excess such as in CAH (18, 19). Prompting the drive towards the development of new therapies such as this to improve the long-term health outcomes of individuals with rare disorders such as CAH and Cushing’s disease (12). Although there is now momentum in the pharmaceutical industry to develop such treatments, including long-acting glucocorticoid therapies, CRH 1 receptor antagonists, as well as MC2R antagonists (12, 15, 20), to date there is no drug that can directly antagonise ACTH or its action in clinical use. The development of such a drug, especially one that is orally active, would have huge clinical benefit in the management of CAH as well as other conditions associated with ACTH excess (disease or treatment related) such as Cushing’s disease, ectopic ACTH syndrome, prostate cancer, obesity, and depression.

In conclusion, this study has discovered a small-molecule antagonist of ACTH stimulation of the human and mouse MC2R-MRAP, with high selectivity for human MC2 amongst human melanocortin receptors, and potential for an initial* in vivo* stage of testing in a mouse model. With further development, such a molecule could become a prototype for novel therapies in CAH and other diseases with ACTH excess.

## Declaration of interest

L F C and A J L C are on the scientific advisory board and consult for OMass Therapeutics. Li Chan and Adrian Clark are part of the Editorial Board for *Endocrine Connections* but have had no involvement with the peer review process of this manuscript. The other authors have nothing to disclose.

## Funding

L F C received funding from International Fund for research on Congenital Adrenal Hyperplasia (IFCAH), British Society of Paediatric Endocrinology and Diabetes (BSPED), Barts and the London Charity (MGU0458), and Medical Research Council http://dx.doi.org/10.13039/501100000265 (MRC) UK/Academy of Medical Sciences http://dx.doi.org/10.13039/501100000691 Fellowship Grant G0802796, 217543/Z/19/Z Wellcome Trust http://dx.doi.org/10.13039/100010269. MRC Collaborative Awards in Science and Engineering (CASE) Studentship Grants MR/J006394/1 (A J L C and L F C) supporting M H and MR/R015686/2442100 (L F C) supporting D P.
